# Supplementary material for: Hydrogen spillover assisted by oxygenate molecules over nonreducible oxides
Source: Nat Commun. 2022 Mar 18;13:1457. doi: 10.1038/s41467-022-29045-6 (PMC8933562; doi:10.1038/s41467-022-29045-6)
Supplement: Supplementary file 1 — Supplementary Information [file 41467_2022_29045_MOESM1_ESM.pdf]

Supplementary Information

Hydrogen spillover assisted by oxygenate molecules over nonreducible oxides

Mingwu Tan, Yanling Yang, Ying Yang, Jiali Chen, Zhaoxia Zhang, Gang Fu, Jingdong Lin, Shaolong Wan, Shuai Wang* & Yong Wang*

*Corresponding authors. Email: shuaiwang@xmu.edu.cn (Shu.W.); yongwang@pnnl.gov (Y.W.)

This supporting information file contains a detailed description of the catalyst synthetic route, and additional data of catalyst characterization and control experiments, which are shown in Supplementary Fig. 1 -16 and Supplementary Table 1.

Table of contents

Supplementary Fig. 1. Synthetic route of the hierarchically structured Pt@-Fe@SiO_2. 2
Supplementary Fig. 2. Size distribution for micropores and macropores of the SiO_2 support. 2
Supplementary Fig. 3. XRD patterns of the SiO_2 support. 2
Supplementary Fig. 4. TEM image of the SiO_2 support 3
Supplementary Fig. 5. N_2 adsorption-desorption results for the prepared catalysts... 3
Supplementary Fig. 6. XRD patterns of the reduced catalysts 4
Supplementary Fig. 7. TEM images and Pt particle size distribution for 0.5Pt@SiO_2 and 2Pt@SiO_2. 4
Supplementary Fig. 8. Effect of H_2 pressure on guaiacol HDO over Fe and Pt-Fe catalysts 5
Supplementary Fig. 9. Catalytic performance of guaiacol HDO over 1Pt@-10Fe@SiO_2 5
Supplementary Fig. 10. Catalytic performance of guaiacol HDO over 1Pt@SiO_2. 6
Supplementary Fig. 11. Comparison of catalytic activity between Fe and Pt-Fe catalysts 6
Supplementary Fig. 12. Effect of NO cofeeding on guaiacol HDO with H_2 directly used as the H source 7
Supplementary Fig. 13. Effect of NO cofeeding on guaiacol HDO with H_2 formed in situ form CH_3OH 7
Supplementary Fig. 14. Effect of additives on the formation rate of BTX over 1Pt@-10Fe@SiO_2 8
Supplementary Fig. 15. DFT-derived H-atom addition energies for C_2-C_3 oxygenates 8
Supplementary Fig. 16. Incluence of oxygenate partial pressure on the formation rate of BTX 9
Supplementary Table 1. Effects of H_2 partial pressure and H source on the formation rate of BTX............9

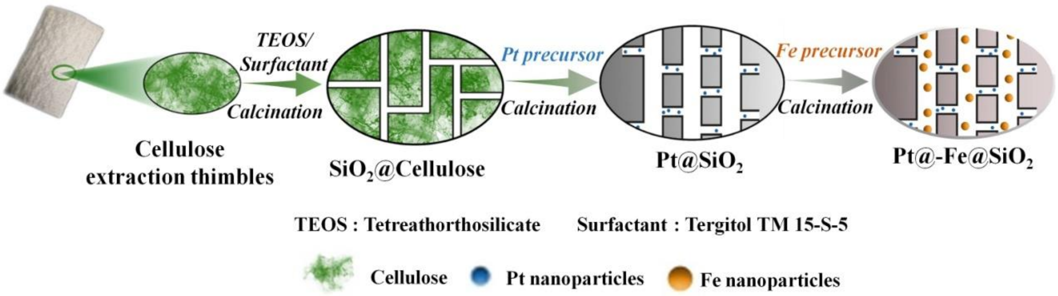

Supplementary Fig. 1 Synthetic route of the hierarchically structured Pt@-Fe@SiO_2 catalysts via a dual template method.

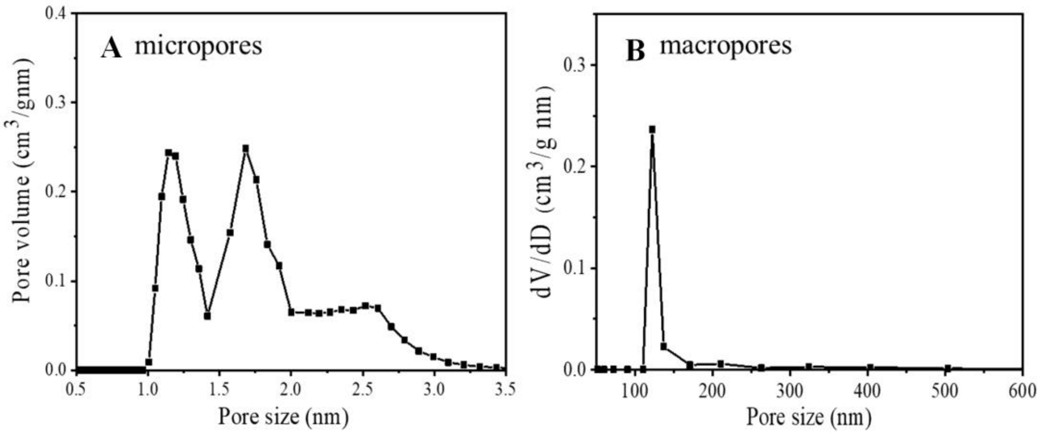

Supplementary Fig. 2 Size distributions for (A) micropores and (B) macropores of the synthesized SiO_2 support. The former is determined via the N_2 physisorption isotherms, while the latter is determined via the mercury intrusion porosimetry.

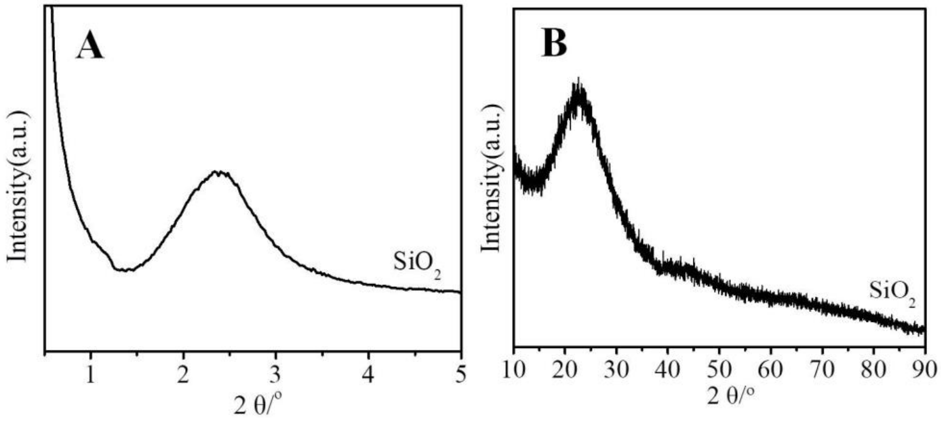

Supplementary Fig. 3 XRD patterns of the synthesized SiO_2 support for (A) the small 2θ range of 0.5˚-5˚ and (B) the wide 2θ range of 10˚-90˚.

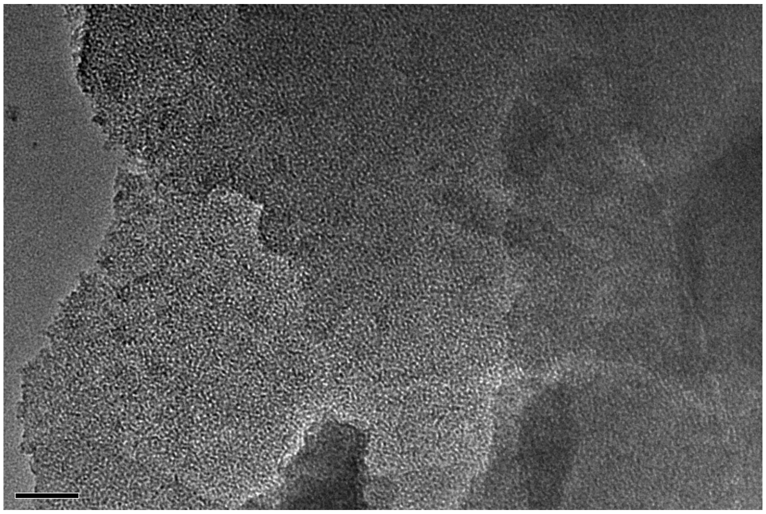

Supplementary Fig. 4 Transmission electron microscopy (TEM) image of the synthesized SiO_2 support.

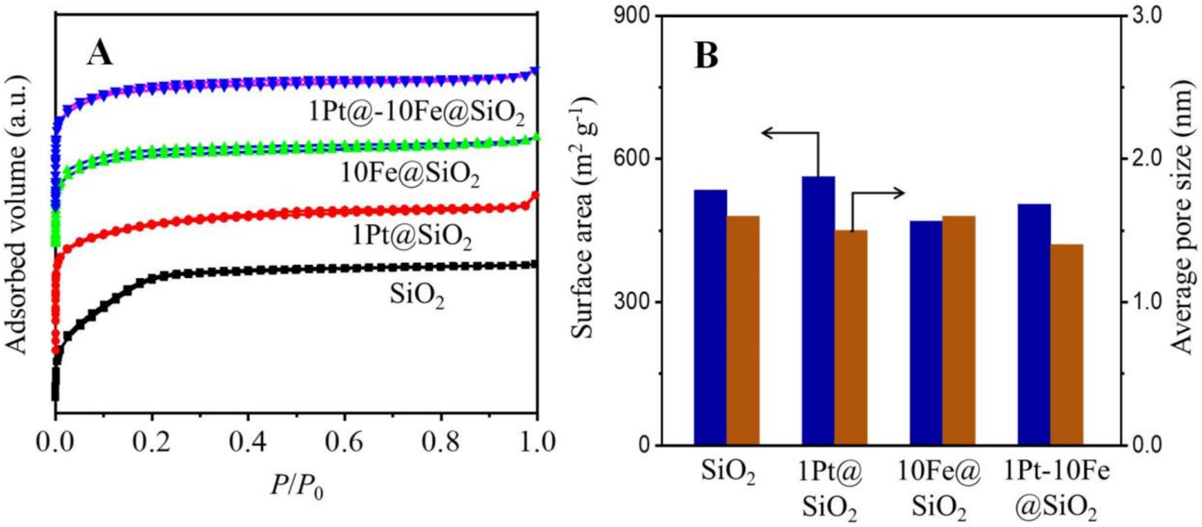

Supplementary Fig. 5 Plots of (A) N_2 adsorption-desorption isotherms and (B) measured values of the Brunauer-Emmett-Teller (BET) surface area and average pore size for SiO_2, 1Pt@SiO_2, 10Fe@SiO_2, and 1Pt@-10Fe@SiO_2.

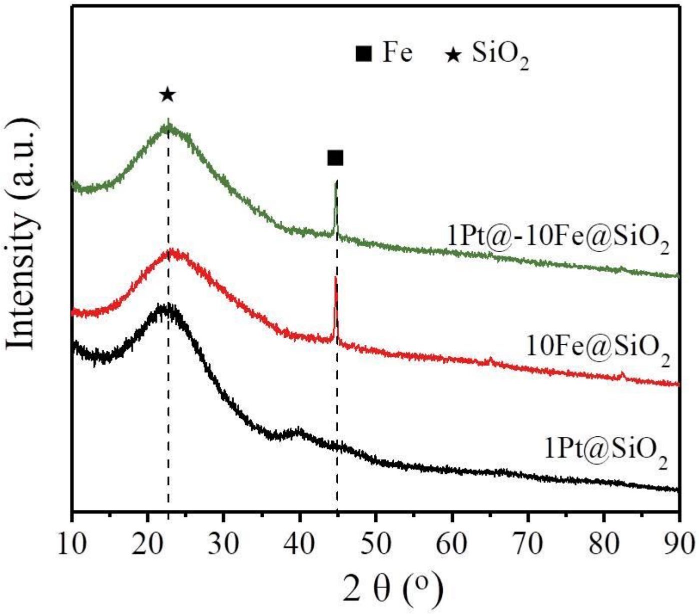

Supplementary Fig. 6 XRD patterns of the SiO_2-supported samples after reduced at 450 ˚C in 50% H_2/N_2 for 2 h.

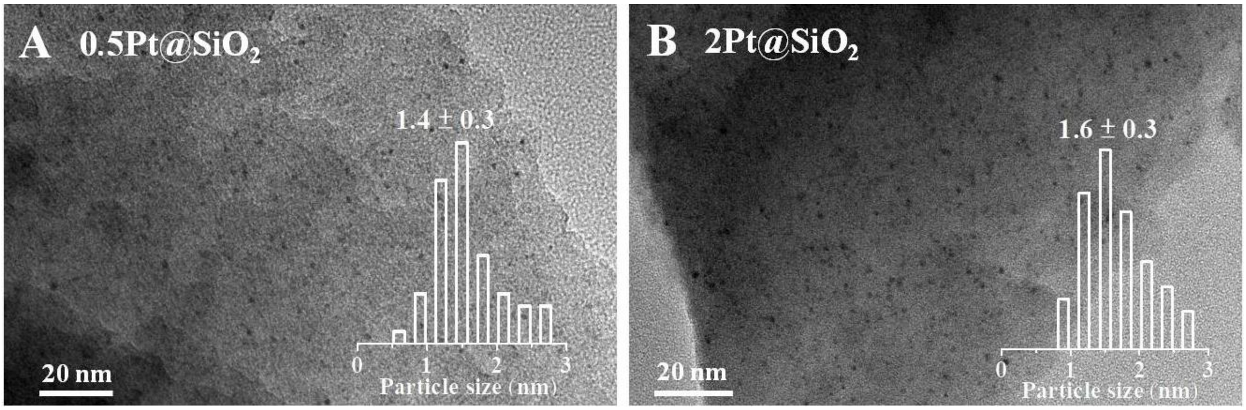

Supplementary Fig. 7 TEM images with corresponding bar charts of statistic Pt particle size distribution for (A) 0.5Pt@SiO_2, (B) 2Pt@SiO_2.

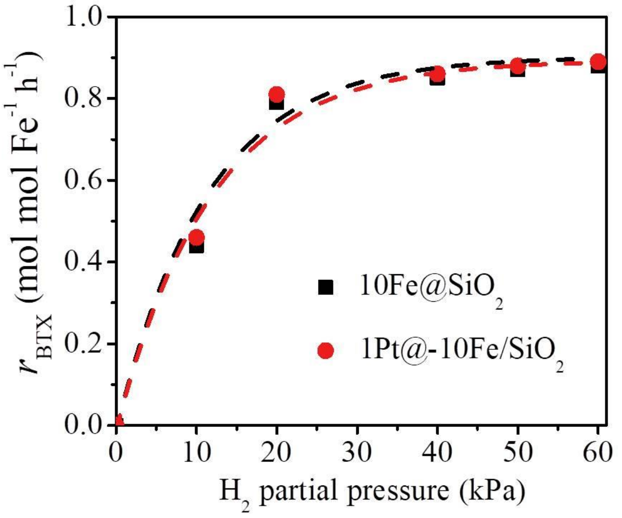

Supplementary Fig. 8 Effect of H_2 pressure on the BTX formation rate (r_{BTX}) of guaiacol hydrodeoxygenation for 1Pt@-10Fe@/SiO$_2$ and 10Fe@SiO$_2$. Reaction condition: 450 ˚C, 0.5 kPa guaiacol, balanced by N_2, 0.25 g.s.mL^{-1} space velocity. Dashed curves indicate trends.

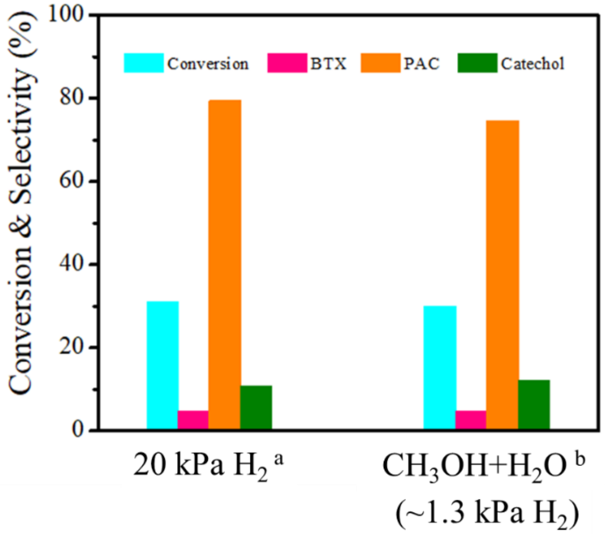

Supplementary Fig. 9 Catalytic performance of gas-phase guaiacol hydrodeoxygenation over 1Pt@-10Fe@SiO$_2$. Reaction condition: 450 ˚C, 0.5 kPa guaiacol, balanced by N_2, 1.25 g·s·mL^{-1} space velocity. [a] Gaseous H_2 (20 kPa) was directly fed into the reactor. [b] CH_3OH (0.5 kPa) and H_2O (0.5 kPa) were cofed into the reactor to generate H_2 in situ via methanol steam reforming (corresponding to an effective H_2 pressure of 1.3 kPa). Here, BTX is denoted for the sum of benzene, toluene, and xylene, while PAC is denoted for the sum of phenol, anisole, and cresol. Note: The guaiacol conversion obtained with cofeeding CH_3OH and H_2O (i.e., ~1.3 kPa H_2) was nearly identical to that of the case with directly feeding H_2 of 20 kPa, reflective of an enhanced HDO activity in the presence of the CH_3OH-H_2O mixture.

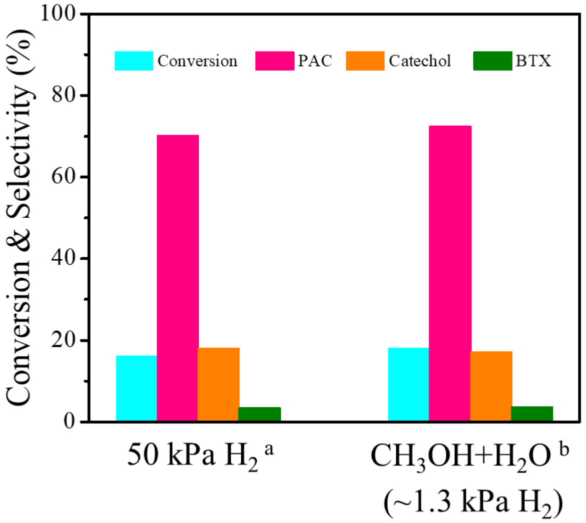

Supplementary Fig. 10 Catalytic performance of gas-phase guaiacol hydrodeoxygenation over 1Pt@SiO_2. Reaction condition: 450 ˚C, 0.5 kPa guaiacol, balanced by N_2, 0.25 g·s·mL^{-1} space velocity. [a] Gaseous H_2 (50 kPa) was directly fed into the reactor. [b] CH_3OH (0.5 kPa) and H_2O (0.5 kPa) were cofed into the reactor to generate H_2 in situ via methanol steam reforming (corresponding to an effective H_2 pressure of 1.3 kPa). Here, BTX is denoted for the sum of benzene, toluene, and xylene, while PAC is denoted for the sum of phenol, anisole, and cresol. Note: The guaiacol conversion obtained with cofeeding CH_3OH and H_2O (i.e., ~1.3 kPa H_2) was very close to that of the case with directly feeding H_2 of 50 kPa, which indicates the HDO activity of 1Pt@SiO_2 can also be enhanced by the CH_3OH-H_2O mixture.

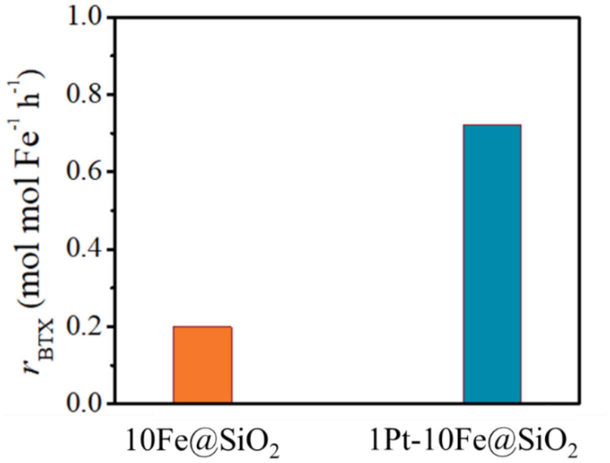

Supplementary Fig. 11 Comparison of the BTX formation rate (r_{BTX}) in guaiacol hydrodeoxygenation between 10Fe@/SiO_2 and 1Pt@-10Fe@SiO_2 when CH_3OH and H_2O were cofed instead of H_2. Reaction condition: 450 ˚C, 0.5 kPa guaiacol, 0.5 kPa CH_3OH, 0.5 kPa H_2O, balanced by N_2, 0.25 g.s.mL^{-1} space velocity.

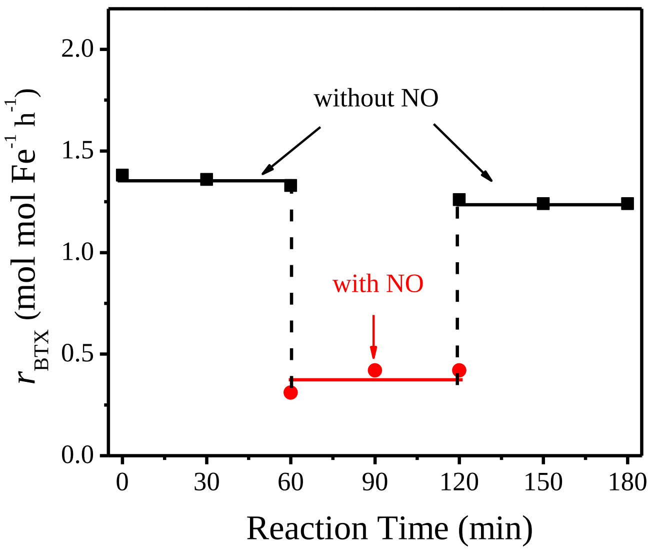

Supplementary Fig. 12 Effect of NO cofeeding on the formation rate of BTX (r_{BTX}) over 1Pt@-10Fe@SiO_2. Reaction condition: 450 ˚C, 0.5 kPa guaiacol, 20 kPa H_2, 0.5 kPa CH_3OH, and cofeeding with or without 3.5 kPa NO, balanced by N_2, 0.25 g·s·mL-1 space velocity.

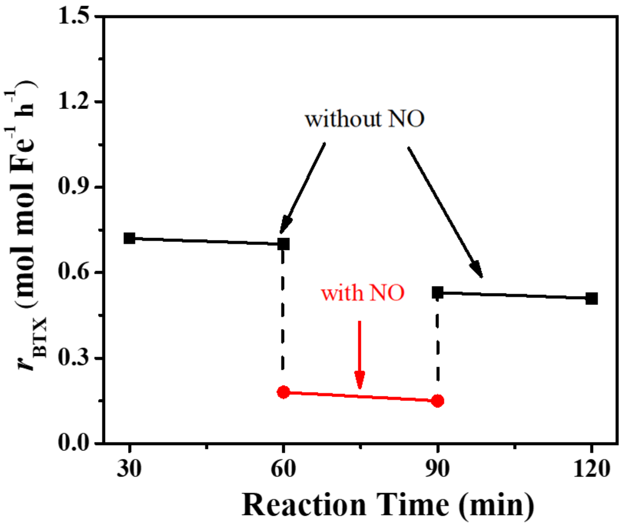

Supplementary Fig. 13 Effect of NO cofeeding on the formation rate of BTX (r_{BTX}) over 1Pt@-10Fe@SiO_2 with H_2 generated in situ from a mixture of CH_3OH and H_2O. Reaction condition: 450 ˚C, 0.5 kPa guaiacol, 0.5 kPa CH_3OH, 0.5 kPa H_2O, and cofeeding with or without 3.5 kPa NO, balanced by N_2, 0.25 g·s·mL^{-1} space velocity. Note: It is observed that the 1Pt@-10Fe@SiO_2 catalyst slightly deactivated along time-on-stream during cofeeding CH_3OH and H_2O, which is plausibly due to the oxidation of metallic Fe by H_2O under low H_2 pressures (*ACS Catal*. 2020, 10, 7884-7893).

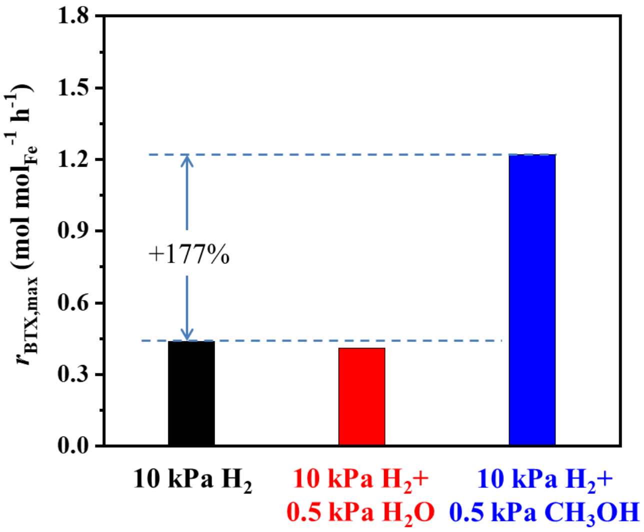

Supplementary Fig. 14 Effect of additive on the formation rate of BTX (r_{BTX}) over 1Pt@-10Fe@SiO_2. Reaction condition: 450 ˚C, 0.5 kPa guaiacol, 10 kPa H_2, 0.5 kPa H_2O or CH_3OH cofed, balanced by N_2, 0.25 g·s·mL^{-1} space velocity.

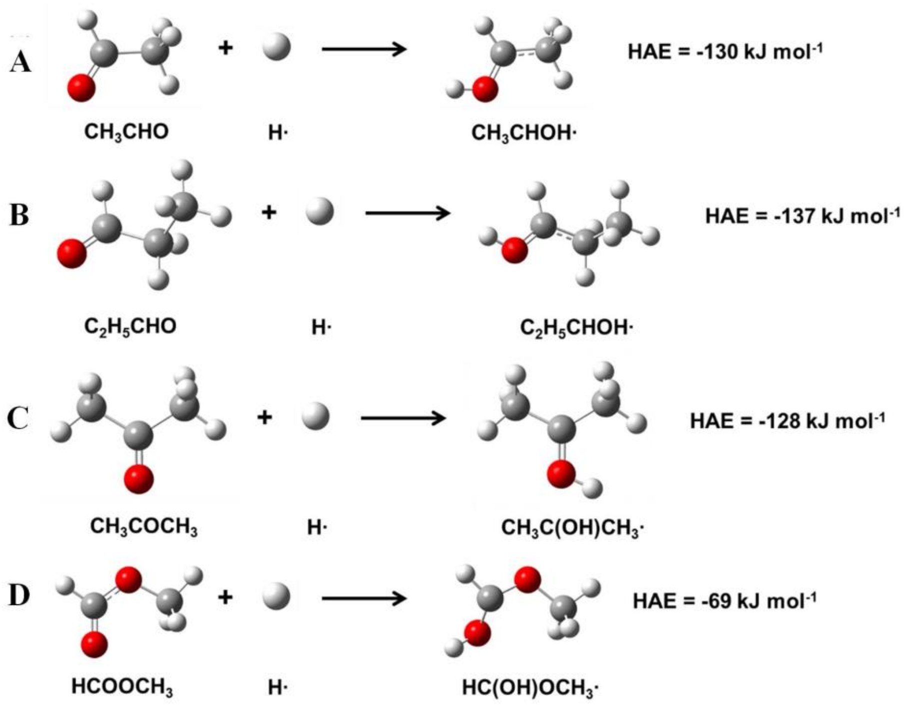

Supplementary Fig. 15 DFT-derived H-atom addition energies (HAE) for (A) acetaldehyde, (B) propanal, (C) acetone, and (D) methyl formate at the B3LY/6-311+G(d,p) level of theory.

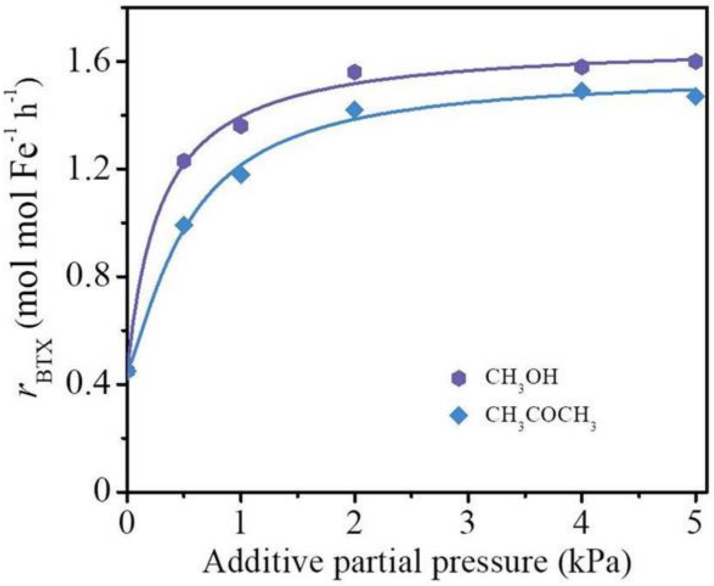

Supplementary Fig. 16 The formation rate of BTX (r_{BTX}) in guaiacol hydrodeoxygenation catalyzed by 1Pt@-10Fe@SiO_2 as a function of the partial pressure of methanol or acetone additive in the presence of 10 kPa H_2. Reaction condition: 450 ˚C, 0.5 kPa guaiacol, balanced by N_2. Solid curves indicate trends.

Supplementary Table 1 Effects of H_2 partial pressure and the source of the H_2 feed on the formation rate of BTX (r_{BTX}) for gas-phase guaiacol hydrodeoxygenation over 1Pt@-10Fe@SiO_2.[a]

Entry	H_2 pressure (kPa)	Guaiacol conversion (%)	Carbon selectivity (%)				Formation rate of BTX (mol mol_{Fe}^{-1} h^{-1})
			Catechol	PAC[b]	BTX[c]	C_1-C_2[d]	
1	10	89.4	5.7	61.2	27.6	< 0.1	0.44
2	50	96.3	2.2	48.1	48.8	< 0.1	0.84
3	1.3 (MSR)[e]	91.0	2.2	46.6	43.8	< 0.1	0.72
4	11 (MSR)[f]	97.2	0.7	22.2	73.2	< 0.1	1.28

[a] Reaction condition: 450 ˚C, 0.5 kPa guaiacol, 10 or 50 kPa H_2, balanced by N_2, 0.25 g·s·mL^{-1} space velocity. [b] Denoted for phenol, anisole, and cresol. [c] Denoted for benzene, toluene, and xylene. [d] Mainly including methane, CO, CO_2, and ethane. [e] Effective H_2 pressure generated in situ via methanol stream reforming (MSR) by cofeeding CH_3OH (0.5 kPa) and H_2O (0.5 kPa) instead of H_2. [f] Cofeeding CH_3OH (4.8 kPa) and H_2O (4.8 kPa) instead of H_2.
